# Beyond Buddhism and animism: A psychometric test of the structure of Burmese Theravada Buddhism

**DOI:** 10.1371/journal.pone.0226414

**Published:** 2019-12-17

**Authors:** Mark Stanford, Jonathan Jong

**Affiliations:** Institute of Cognitive and Evolutionary Anthropology, University of Oxford, Oxford, United Kingdom; University of Lleida, SPAIN

## Abstract

Anthropologists and religious scholars have long debated the relationship between doctrinal Theravada Buddhism, so-called ‘animism’, and other folk practices in southeast Asian societies. A variety of models of this relationship have been proposed on the basis of ethnographic evidence. We provide the first psychometric and quantitative evaluation of these competing models, using a new scale developed for this purpose, the Burmese Buddhist Religiosity Scale. Having tested existing hypotheses in our first study (n = 2285) we formulated an alternative model, which was then tested in our second study (n = 3377). We argue that this model provides support for a two-dimensional distinction between great and little traditions, shedding light on decades-old theoretical debates. Far from being in conflict, the transnational religious tradition of the literati and the variegated religious practices of locals appear to be reflected in two complementary dimensions of religiosity. This distinction has been heretofore neglected in psychometric research, but arguably merits attention beyond Buddhism, in the psychology of religion more generally. Our findings suggest that, insofar as research on religiosity relies on doctrinal pronouncements denigrating little traditions as mere superstition, it may be blinded to a crucial dimension of religious life.

## 1. Introduction

In recent years, the psychological study of religion has become more closely integrated with cross-cultural concerns, in particular as it has become increasingly interlinked with cultural evolutionary approaches [1]. But for the psychology of religion to be fit for purpose for cross-cultural research, it has become clear that its methods must begin to move away from those developed exclusively for the study of ‘WEIRD’ populations [2]; the range of measures must be expanded, in particular, for the study of non-Abrahamic religions.

One such context is that of Theravada Buddhism, a religious formation which has given rise to rich debates in anthropology and religious studies, but to which relatively little psychological attention has been paid in recent years. In Burma, a recent reopening of possibilities to conduct research provides the opportunity to revisit key debates surrounding not only the structure of Burmese Buddhism, but also the nature of Theravada Buddhism more widely. These debates have long informed broader theories of religion, and whether and how it can be decomposed into ‘great’ and ‘little’ traditions [3–5]. But while some of the salutary studies informing those theories originated in Burma, five decades of dictatorship made it nearly impossible for researchers to revisit the Burmese case. Today, it is once again possible to conduct research in Burma, and a growing number of anthropologists and other social researchers are reassessing Burmese Buddhism, and its implications for our understanding of religion more widely.

The present study was motivated not only by the need to fill the gap in cross-cultural measures of religiosity, but also by the prospect of using psychometric evidence to evaluate the adequacy of several competing models of the nature of Burmese Buddhism, and Theravada more generally. In so doing, it provides the first quantitative and psychological test of these models, many of which were formulated on the basis of qualitative research conducted several decades ago.

### 1.1 Concepts of Theravada religion

While lacking a single, centralised authority, Theravada Buddhism—as practised predominantly throughout Southeast Asia and Sri Lanka—presents itself as a transnational religious community, characterised by a constant flow and interchange of people, texts, and ideas [6]. This has led to a degree of consistency across localities in the overarching cosmology and value system of Theravada monks and other expert practitioners. In particular, starting in the late 18^th^ century, but accelerating markedly in the mid-20^th^ century, Theravada has witnessed the spread of ‘revivalist’ or ‘protestant’ [7–9] movements which promote a purified form of the religion, comprising mass meditation, lay textual study, and an ostensible return to an austere, practical form of Buddhism, often presented as ‘scientific’ or ‘modern’ [10]. While these movements likely typify longstanding concerns with the perennial decline of the Buddhist community, and therefore cannot necessarily be seen as a simple rupture caused by colonialism [11,12], they have nevertheless been tightly interwoven with 20^th^ century political concerns, and adopted at the core of nationalist ideologies from Burma to Sri Lanka, in which post-colonial states present themselves as representatives and protectors of orthodoxy [13–15].

At the same time, transnational Theravada doctrine invariably coexists with a vast range of other local practices and beliefs, including various forms of spirits, demigods, witches and alchemists, as well as magic and divination. Far from being merely tolerated by Buddhism, these elements are typically highly integrated into the Buddhist system, given space for shrines within Buddhist pagodas, and mentioned in mythological accounts of the life of the Buddha. Nevertheless, the official religion, particularly in its ‘revivalist’ forms, is often indifferent to them, and at worst sees them as an unfortunate remnant of pagan times, clung to by laypeople only out of ignorance [16]. In this uneasy relationship, doctrine often asserts that while Buddhism is a ‘religion’ (in Burma, a concept only introduced during the 19^th^ century colonial encounter [17]), local elements are mere ‘tradition’—cultural ephemera of no particular importance.

Outside observers have long debated how best to understand the relationship between these two sides, and in so doing, have arguably been heavily influenced by modern Theravada’s own preoccupation with denigrating local practices in favour of doctrinal orthodoxy [18]. In Burma, colonial scholars tended to endorse the view that Buddhism and so-called ‘animism’, or the folk religion revolving around the propitiation of local spirits known as *nats*, were two distinct religions; and that Buddhism, having unsuccessfully attempted to supplant the older animistic religion, formed nothing but a ‘thin veneer’ over animism for the majority of the populace [19]. Indeed, the two religions were often seen as in direct conflict; animism was antagonistic to the teachings of Buddhism, but nevertheless remarkably resilient to official attempts to stamp it out [20].

Amongst anthropologists, the most elaborate defence of the ‘two religions’ view was articulated by Spiro [21], who rejected the claim that animism was nothing but a veneer, but argued that Buddhism and the nat cultus indeed comprised two, conflicting religions, coexisting not only in the same society, but in the same individuals. For Spiro, while Buddhism enjoyed primacy over animism, the two represented conflicting Apollonian and Dionysian value systems and thus personal orientations, giving rise to an inner conflict amongst practitioners. Similarly, while rejecting the ‘two religions’ view, Mendelson [22] cast Buddhism and animism as occupying two opposing poles of a single continuum of Burmese religion. Thus whether two religions or two parts of one, Theravada and local folk practices were described as intrinsically conflicting.

Against this view, others have long argued that Buddhism and animism form two inseparable parts of a single religious system. One line of argument here is that the two are conceptually interdependent; Buddhism is defined in terms of animist concepts and values, or the reverse, thus it is nonsensical to treat them as conflicting cosmological or value systems [23,24]. Others have gone further, rejecting on structural-functionalist grounds the notion that locally realised Buddhism can be divided into ‘great’ and ‘little’ traditions; on this view, local religion must be treated as a single totality, in which local spirits play a central role [25–27]. In spite of apparent tensions, then, this view suggests we should see Theravada Buddhism as constituting not a bipolar continuum, but a single, unipolar construct.

A final view is that doctrinal Buddhism and animism can be distinguished, but that instead of being in conflict, they fall into two distinct and complementary niches. Here, Buddhism is concerned with ‘otherworldly’ goals of better rebirth and spiritual liberation (the attainment of nirvana), while animism is concerned with ‘worldly’ aims of bettering one’s lot in the present lifetime [28]. One meditates and donates to the monkhood in order to further one’s otherworldly progress; but for more material needs, one must turn to gods, spirits, magic and astrology. On this view, Buddhism and animism should be seen as two dimensions of Theravada religion—‘not one continuum, but two intersecting ones’ [29].

In addition to these three characterisations of the relationship between Buddhism and animism, the picture is still further complicated by claims for other ways of breaking down the religious system. Spiro [30] claimed that ‘exoteric’ Buddhism comprised three distinct systems: ‘apotropaic’, concerned with bettering one’s lot in the present lifetime; ‘kammatic’, concerned with merit-making in order to secure a better rebirth in the next life; and ‘nibbanic’, concerned with transcending the cycle of rebirth entirely through practices such as meditation, aimed at realisation of the non-reality of the self. Similarly, Ames [29] breaks down Buddhism into a ‘little tradition’, concerned with merit-making for the next life, and a ‘great tradition’, concerned with meditation and transcendence. On these views, then, it is not enough to distinguish Buddhism from folk religion; Buddhism itself comprises distinct religious systems, each with its own objectives and values.

Moreover, the Burmese case presents a further puzzle still. For beyond the simple dichotomy of Buddhism and nat propitiation, Burmese religion also comprises a third branch: that of the *weikza*, a term variously translated as wizards or alchemists. Followers of weikza sects fit squarely within the soteriological goals of Buddhism, but rather than offering enlightenment through meditation, the weikza path provides them with the possibility of using magical and alchemical means to achieve virtual immortality, prolonging life until the appearance of the next Buddha, thus enabling enlightenment at that time [31]. In the short term, weikza practitioners seek to master magical energy flows for other purposes, too. While Spiro [30] characterises the weikza cults as ‘esoteric Buddhism’, a grafting together of otherwise contradictory Buddhist and magical beliefs, Mendelson [22] places the weikza at the centre of his continuum stretching from Buddhism to folk belief. Meanwhile, more recent anthropology has taken issue with the characterisation of the weikza as merely a marriage of high Buddhism and folk tradition, pointing out the deep cosmological and affective interdependency of the weikza cult and other domains of the religious system as a whole [32].

While these debates may appear parochial, they go to the heart of global questions of how to conceptualise aspects of religion more broadly. In anthropology, these begin with Redfield [3], who suggested that the ‘great tradition’ of the literati and the ‘little tradition’ of folk religion comprised two coexisting strata of local religion. While positions like those of Spiro [21] and Nash [28] are compatible with this view, others, including those of Obeyesekere [26] and Tambiah [33], would suggest that the distinction must be re-evaluated: local religion is one, integrated totality, and if there is such a thing as a ‘great tradition’, it is that which exists in urban centres and specialised institutions, not as a stratum of village life. In the study of ancient Near-Eastern religions, the distinction appears, no less controversially, as that between ‘official’ and ‘popular’ religions [34]. Ostensibly great and little traditions are found worldwide; but the Theravada case provides an important test for a conceptual distinction which, according to many of its proponents, has universal import.

Research on Burmese Buddhism has continued to explore the conceptual interdependencies between Buddhism and animism, calling into question the possibility of treating the two as distinct religions [18,35]. However, even if this is so, it leaves open the question of whether they can be distinguished as components of the same religion, and if so, what sort of relationship holds between them.

### 1.2 Psychometric measures of religiosity

The psychology of religion offers a novel lens through which to examine these questions. Rather than examining religion as a cultural system, psychology has tended to study spirituality, religiosity, or religious orientation as individual traits. Individual religiosity is not equivalent to the institutions, social structures and shared meanings which might be taken to constitute ‘religion’, and indeed, critics have argued that the psychology of religion has focused perhaps too narrowly on individual traits [36]. Nevertheless, individual orientations toward religious systems may provide valuable insight into the nature of the religious systems themselves—not least because if a multitude of religious systems are present, then individuals may be expected to display differing orientations toward each. Thus psychometric assessment of religiosity offers a novel individual-level tool to address social and cultural debates.

However, methods for measuring religiosity leave something to be desired. In general, robust areas of psychometrics might be hoped to converge around an agreed set of measures corresponding to constructs of interest. Religiosity scales, on the other hand, have continued to proliferate over decades, with 126 catalogued in Hill and Hood [37], and many more added since [38]. On the one hand, this has resulted from a range of different interests on the part of researchers—from levels of religious commitment, to attitudes toward God, to the use of religion as a coping mechanism. But on the other hand, a lack of agreed theoretical frameworks has hampered consensus about the nature of the objects of measurement [39].

Much theoretical attention has been paid to whether religiosity has a universal underlying structure. Allport [40,41] argued that individuals fell on a spectrum of intrinsic versus extrinsic religious orientation, concerned with spiritual pursuit for its own sake as compared with religiosity for the sake of obtaining other benefits, such as social status. To these two religious orientations have been added further candidates, such as ‘quest’, a construct designed to capture the late 20^th^ century growth of spiritual seeking outside of orthodoxy [42]; and religious fundamentalism as a distinct orientation [43]. Glock and Stark [44] suggested breaking down religiosity into a five-dimensional construct, encompassing experiential, ritualistic, ideological, intellectual and consequential religiosity spectra. Since then, there has been a proliferation of rival multidimensional constructs [37].

While there is, then, an overabundance of measures, the vast majority of these have been developed for use in ‘WEIRD’ populations, especially Christian ones [45]. Many scales consist of highly culturally specific items; and even the constructs in question may be culturally specific. For example, scales focused on beliefs may be less relevant in orthopraxic religious traditions, while those focused on attitudes to God are of questionable relevance in polytheistic or nontheistic religions. The development of ecologically valid measures, let alone cross-culturally applicable ones, has barely begun.

In the case of Buddhism, there are only a handful of extant scales. The 11-item Buddhist Beliefs and Practices Scale (BBPS) was developed to measure religiosity in a study of the effects of meditation retreats in Thailand [46]; the content of this scale has not been made freely available. Thanissaro’s 24-item Scale of Attitudes toward Buddhism (TSAB) [47,48] was developed to measure attitudes toward Theravada beliefs and practices in the UK. Finally, the Buddhist Coping Scale (BCOPE) measures the reliance of American Buddhists on Buddhist coping mechanisms in times of difficulty [49–51]; it is an adaptation of the more general Religious Coping Scale [52]. All three of these scales contain only items reflecting doctrinal Buddhism, with no mention of elements of folk religion, ‘animism’, astrology and the like; thus they are not suitable for the purpose of evaluating competing hypotheses about the structure of a religious system which may include these elements.

Against this, it might be argued that animism is not religion at all, but instead a form of spirituality. Much of the psychometric literature has coalesced around a distinction between the two, in which ‘spirituality’ is often used to indicate ‘the search for the sacred’, including within ‘non-traditional contexts’, while ‘religion’ is taken as ‘established institutional contexts designed to facilitate spirituality’ [39]. Thus distinct measures of religiosity and spirituality have been developed; and it has even been argued that spirituality constitutes a universal aspect of human experience, detectable in a wide range of populations [53,54]. In Burma, Buddhism is typically referred to as a ‘religion’, while animism and other related practices are often given a label like ‘spirituality’. Thus it might be tempting to suggest that existing Buddhism scales are adequate, because animism requires not a measure of religiosity, but one of spirituality.

However, both doctrinal Buddhism and folk practices include elements of both religion and spirituality, as defined in the psychometric literature. Theravada practitioners meditate within established institutional contexts, with a mind to the development of their own moral capacities; but they also often report experiencing transcendent, altered states during the practice of meditation [55]. Meanwhile, both nat devotees and followers of the weikza practise within a clear institutional structure, characterised by sects, hierarchical divisions, and regular meetings and festivals which help to standardise beliefs and practices [18]. In spite of the official discourse, which tends to deride animism as not constituting a religion, the distinction between doctrinal Buddhism and other elements of Burmese religion does not coincide with the distinction between religion and spirituality, as it has been made by psychometricians.

For these reasons, in order to provide an adequate religiosity measure for use in psychological research in Burma, it was necessary to develop a new scale of Burmese Buddhist religiosity, which would include elements representative of the varied aspects of religion and spirituality practised by those Burmese people who call themselves Buddhists. Rather than assuming a priori that one or another aspect might be omitted by virtue of constituting a distinct system, we aimed to construct a scale which would measure engagement with nats, weikza, and astrology, as well as with doctrinal Theravada.

By constructing such an inclusive scale, we aimed to enable the use of factor analysis to reveal the underlying structure of Burmese Buddhist religiosity, and thus to evaluate competing models of that structure, as outlined in 1.1 above—testing firstly, claims that the religion constitutes one or two systems; and secondly, claims that if there is more than one religious system present, those systems are complementary or in conflict.

## 2. Scale construction and pilot studies

The Burmese Buddhist Religiosity Scale (BBRS) was constructed with two criteria in mind: First, it should include items reflecting a variety of Burmese religious forms, including doctrinal Buddhism, the nat cult, astrology and the weikza line; and second, it should allow for the capture of multiple broad dimensions of religiosity which have been identified cross-culturally. For the latter purpose, we adapted the five-dimensional Centrality of Religiosity Scale (CRS), an instrument designed for cross-cultural and interreligious studies, which captures multiple key distinctions in the psychometric literature, and which has been deployed in more than 100 studies across 25 countries [56]. For each of the five CRS dimensions, items were included, where possible, corresponding to doctrinal Buddhism, nat propitiation, and the weikza line. A total of 30 items were employed, including 21 frequency items, 3 interest items, 4 belief items, and 2 extrinsic items (see Table 1).

**Table 1:** Items corresponding to five CRS dimensions and three religious divisions.

	Doctrinal Buddhism	Nat cult	Weikza line
*CRS Public*	How often do you visit a pagoda to observe the Sabbath or the cycles of the moon, or take part in other Buddhist ceremonies? How often do you give alms (food, money, or other donations) to monks? In total, how much time in your life have you spent as a monk or nun? *(In years*, *months*, *and weeks*?*)* How often do you visit or consult with an astrologer?	How often do you take part in nat festivals, nat ceremonies, or ceremonies to communicate with spirits? How often do you visit or consult with a spirit medium?	How often do you visit or consult with a weikza practitioner?
*CRS Private*	How often do you pray to the Buddha or make offerings to statues of the Buddha? How often do you meditate? How often do you visit a pagoda to pray, meditate or make offerings to the Buddha?	How often do you make offerings or pray to nats, bobogyi or thaik? How many images or statues of nats do you have in your home? How often do you visit a nat shrine or pagoda to make offerings to the nats?	How often do you make offerings or pray to weikza or weikza zawgyi?
*CRS Religious experience*	How often do you experience situations in which you have the feeling of anatta (non-self)? How often do you experience situations in which you have the feeling that the Buddha wants to communicate or reveal something to you?	How often do you experience situations in which you have the feeling that nats, bobogyi or thaik intervene in your life? How often do you experience situations in which you have the feeling that nats, bobogyi or thaik want to communicate or reveal something to you?	How often do you experience situations in which you have the feeling that weikza or weikza zawgyi intervene in your life? How often do you experience situations in which you have the feeling that weikza or weikza zawgyi want to communicate or reveal something to you?
*CRS Ideology*	To what extent do you believe in reincarnation? To what extent do you believe events in your life are determined by karma?	To what extent to you believe in the existence of nats, bobogyi, or thaik? To what extent do you believe events in your life are determined by nats or other spirits?	
*CRS Intellect*	How interested are you in learning more about Buddhism? How often do you think about Buddhism or the teachings of the Buddha?	How interested are you in learning more about the nats, bobogyi, or thaik? How often do you think about the nats, bobogyi or thaik?	How interested are you in learning more about weikza or weikza zawgyi? How often do you think about the weikza?

The first draft of the BBRS was translated into Burmese by a professional translator, and back-translated into English. Interviews were conducted with informants in Yangon in June 2017, which led to further adjustment of the scale items.

An online pilot study was carried out in August 2017 (n = 456). Participant feedback collected via Facebook suggested that the translation quality was in fact very poor. Working with two further translators, a revised version was produced, which was piloted online in September 2017 (n = 2283). This time, feedback indicated fewer translation problems. The translators were then consulted once again to produce the final Burmese version of the BBRS.

In order to assess the relationship between the BBRS and a measure designed for doctrinal Theravada only, Thanissaro’s Scale of Attitudes to Buddhism (TSAB) [47] was also translated into Burmese and validated in the same way.

## 3. Study 1

Rather than posit a single hypothesis, we set out to test a number of competing hypotheses, corresponding to the various sides of the anthropological debate outlined in 1.1 above. The competing hypotheses were as follows:

- One system: Doctrinal Theravada, nat propitiation, weikza adherence and astrology form a single, integrated religious system. Thus individual religiosity consists of a single, one-dimensional construct.
- Two systems, conflicting: Buddhism, including weikza practices and astrology, and nat worship, form two distinct and conflicting systems. Thus religiosity is best captured by a two-factor solution, in which the two factors negatively covary.
- Two systems, complementary: Buddhism, including weikza practices and astrology, and nat worship, form two distinct, but complementary, systems. Thus religiosity is best captured by a two-factor solution, in which the two factors positively covary.

### 3.1 Methods

Participants were 2285 adult Facebook users in Myanmar who identified themselves as Buddhists, and identified Burmese as their mother tongue. Those who did not meet these criteria, or who failed to complete the religiosity scale were excluded from the original sample (n = 5639). The religiosity scale, along with demographic questions, was administered as part of a larger questionnaire including moral psychology questionnaires administered first. This sample skewed heavily toward university-educated respondents.

Table 2 shows sample characteristics of both study 1 and study 2, compared with population traits taken from the 2014 census [57] and, for income, from Nielsen MMRD [58].

**Table 2:** Sample characteristics.

	Population	Study 1	Study 2
Sample size	N/A	2285	3377
Male/female sex ratio	.93	1.43	1.18
Median age	27	29	32
Mean monthly income (In 1000s of Myanmar kyat)	250	457	390
% completed post-secondary education	10.34%	85.7%	60.1%
% urban	29.59%	88.4%	58.9%

Ethics approval for studies 1 and 2 was obtained from the University of Oxford School of Anthropology and Museum Ethnography Departmental Research Ethics Committee. All participants completed an online informed consent form prior to participation.

### 3.2 Results

CFA, EFA and SEM were carried out in Mplus 8 [59]. All analyses were carried out using sample weights calculated to reflect the age and sex distribution in the 2014 Myanmar census [57]. Weights were generated using the anesrake package for R [60], using recommended parameters [61,62]. Calculations for both CFA and EFA were made using the weighted least squares method with mean and variance-adjusted chi-square (WLSMV). Likert scales were treated as ordinal variables in all models. Comparative Fit Index (CFI) and Root Mean Square Error of Approximation (RMSEA) are reported below, using generally accepted rules of thumb such that adequate model fit is indicated by CFI ≥ .95 and RMSEA ≤ .06 [63].

#### 3.2.1 Confirmatory factor analysis

A one-factor CFA model including all items showed poor fit (CFI = .766, RMSEA = .092; χ^2^ (405, *N* = 2285) = 8183.090, p < 0.001; TLI = .749), leading us to reject hypothesis (A) of a single, unipolar construct.

To test our remaining two hypotheses, that Buddhism and animism represent two systems, either conflicting (hypothesis B) or complementary (hypothesis C), a two-factor model was fitted, with nat-related items loading on one factor, and all other items (including weikza items) on the other factor. This model, too, showed a poor fit (CFI = .764; RMSEA = .093; χ^2^ (404, *N* = 2285) = 8406.616, p < 0.001; TLI = .746).

An alternative version of these hypotheses would be to relax the assumption that the weikza cult forms part of ‘Buddhism’. To test this, a three-factor model was fitted, separating Buddhist, animist, and weikza items; this model was unsatisfactory (CFI = .885; RMSEA = .065; χ^2^ (403, *N* = 2285) = 4324.228, p < 0.001; TLI = .875). Thus hypotheses (B) and (C) were rejected.

#### 3.2.2 Exploratory analysis

**Fig 1:**
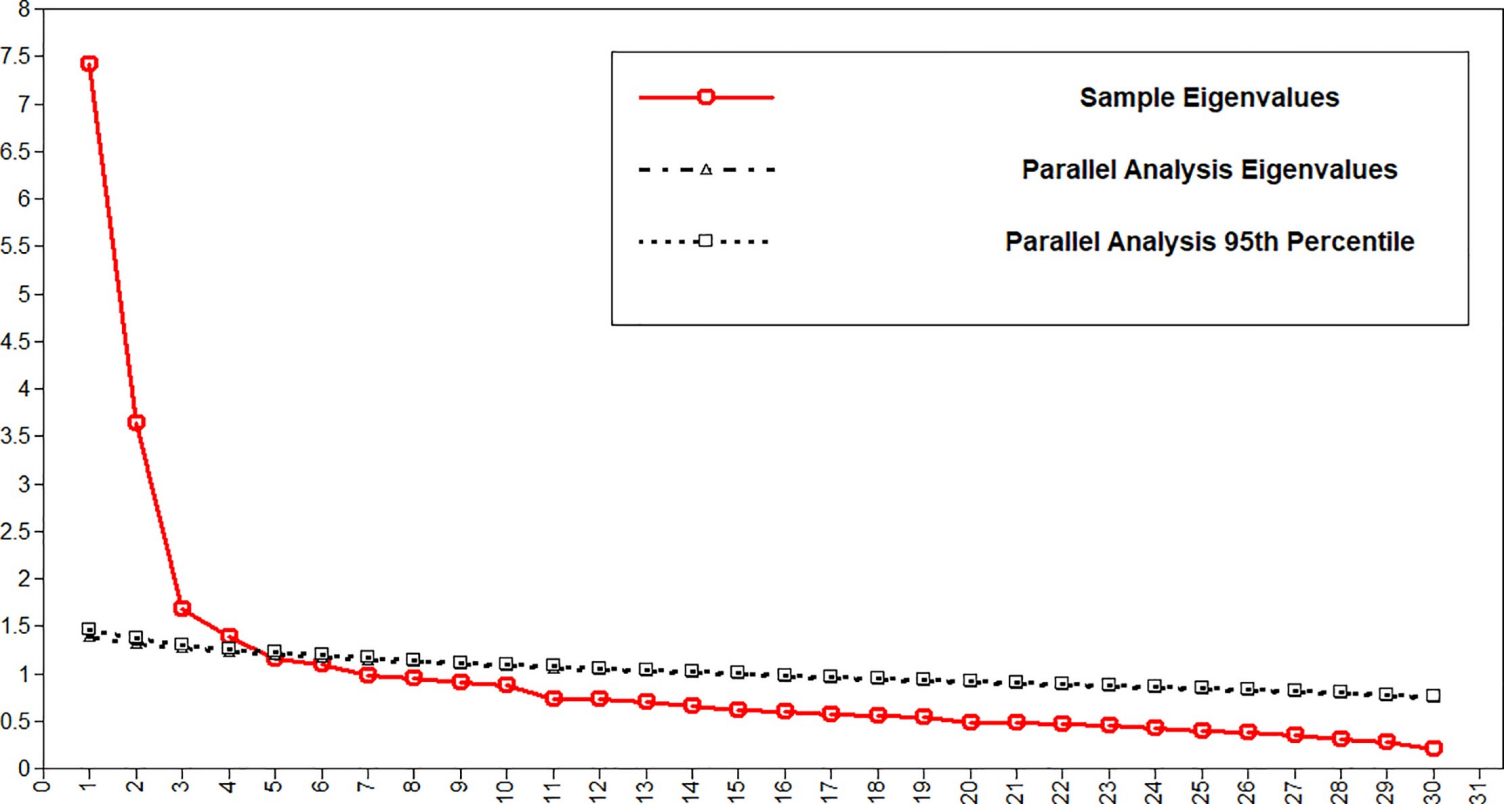
EFA parallel analysis.

As previously hypothesised structures did not fit the data, EFA was performed in order to determine whether any other meaningful structure might be present. Parallel analysis [64], carried out in Mplus 8, suggested a four factor solution (Fig 1). Velicer’s [65] MAP test, carried out with polychoric correlations in the paramap package for R [66], suggested only two factors, as did the Very Simple Structure procedure [67], performed using the R psych package [68]. Meanwhile, visual inspection of the scree plot of sample eigenvalues [69] suggested a two factor solution. Given the tendency of parallel analysis to overestimate slightly the number of factors [70], a two or three factor solution appeared most likely to be acceptable.

While EFA fit indices improved markedly from a one-factor (CFI = .766; RMSEA = .092; χ^2^ (405, *N* = 2285) = 8183.114, p < 0.001; TLI = .749) to a two-factor (CFI = .912; RMSEA = .058; χ^2^ (376, *N* = 2285) = 3297.096, p < 0.001; TLI = .898) solution, they increased less substantially with a third factor (CFI = .960; RMSEA = .041; χ^2^ (348, *N* = 2285) = 1662.065, p < 0.001; TLI = .951). Nevertheless, as the third factor increased fit parameters to acceptable thresholds, and loadings appeared to be interpretable, we interpret all three factors below.

Factor loadings with orthogonal and oblique (both GEOMIN) rotations are presented in Table 3 Given the similarity of the loadings, and the low correlation between factors in the oblique solution, it would appear that these three factors may be taken as more or less orthogonal. As time spent as a monk failed to load with sufficient magnitude on any factor, it was removed from subsequent analyses.

**Table 3:** EFA loadings and factor correlations with GEOMIN rotation (*: p < .05; loadings > .200 in bold).

	Orthogonal (GEOMIN)			Oblique (GEOMIN)		
	Factor 1	Factor 2	Factor 3	Factor 1	Factor 2	Factor 3
*Frequency of weikza intervening in life*	0.860*	0.079	-0.144*	0.831*	-0.004	-0.170*
*Frequency of praying to weikza*	0.860*	0.028	0.041	0.869*	-0.038	0.017
*Frequency of praying to nats*	0.845*	-0.065	0.077*	0.868*	-0.128*	0.048*
*Frequency of nats intervening in life*	0.843*	0.101	-0.130*	0.815*	0.022	-0.153*
*Frequency of thinking about nats*	0.832*	-0.012	0.055	0.847*	-0.075*	0.03
*Frequency of visiting a weikza practitioner*	0.831*	-0.044	0.018	0.843*	-0.111*	-0.01
*Frequency of communicating with weikza*	0.830*	0.131	-0.182*	0.790*	0.048	-0.204*
*Frequency of communicating with nats*	0.816*	0.092	-0.198*	0.776*	0.007	-0.223*
*Frequency of thinking about weikza*	0.798*	0.086	0.078	0.809*	0.03	0.061*
*Frequency of making offering to nats*	0.686*	-0.092	0.001	0.697*	-0.150*	-0.026
*Frequency of nat ceremonies*	0.669*	-0.088	-0.016	0.677*	-0.146*	-0.043
*Frequency of consulting natkadaw*	0.662*	-0.112*	-0.019	0.671*	-0.170*	-0.047
*Interest in nats*	0.644*	-0.023	0.642*	0.767*	-0.013	0.637*
*Interest in weikza*	0.628*	-0.001	0.638*	0.749*	0.01	0.634*
*Belief in nat influence*	0.565*	-0.104*	0.043	0.584*	-0.148*	0.02
*Frequency of communication with Buddha*	0.517*	0.163*	-0.169*	0.475*	0.108*	-0.179*
*Belief in nats*	0.515*	0.135*	0.239*	0.551*	0.119*	0.237*
*Frequency of consulting astrologer*	0.512*	-0.046	0.016	0.521*	-0.087*	-0.002
*Number of nat images at home*	0.450*	-0.148*	0.078*	0.478*	-0.180*	0.057
*Frequency of praying at pagoda*	0.198*	0.628*	-0.13	0.124*	0.611*	-0.101*
*Frequency of praying to Buddha*	0.190*	0.648*	-0.04	0.130*	0.641*	-0.007
*Frequency of attending pagoda festivals*	0.144*	0.561*	-0.129*	0.075	0.547*	-0.102*
*Frequency of giving alms to monks*	0.129*	0.562*	-0.145*	0.056	0.548*	-0.119*
*Belief in karma*	-0.080*	0.497*	0.488*	-0.031	0.561*	0.533*
*Frequency of meditation*	0.074	0.600*	-0.099	0.006	0.595*	-0.067
*Frequency of thinking about Buddha*	0.052	0.752*	0.071	0.004	0.769*	0.117*
*Belief in reincarnation*	0.017	0.457*	0.513*	0.075	0.514*	0.553*
*Frequency of feeling non-self*	-0.001	0.596*	0.03	-0.044	0.610*	0.067
*Interest in Buddhism*	-0.009	0.680*	0.194*	-0.029	0.712*	0.241*
*Time spent as monk or nun*	0.03	0.070*	0.009	0.026	0.070*	0.012
*Factor 1 correlation*				1.000		
*Factor 2 correlation*				0.167*	1.000	
*Factor 3 correlation*				-0.171*	-0.180*	1.000

The first dimension in our analyses includes multiple elements which cannot obviously be described merely as ‘animism’. While it does load on nat-related practices and beliefs, it also includes both astrology and the weikza line. Both of these are classified as part of Buddhism, not ‘supernaturalism’ or animism, by Spiro [30]. Indeed, weikza concepts, and the value system surrounding them, are thoroughly embedded in Buddhist cosmology [32]. Moreover, this dimension includes one item which is entirely concerned with the Buddha, i.e. the frequency of feeling that the Buddha wants to communicate something to one—an item which surely cannot be neatly labelled as ‘animism’.

While it would also be inappropriate to label the second factor ‘Buddhism’—indeed, that label would be better reserved for the whole, multi-dimensional construct—this dimension does appear to correspond more closely to what might be called ‘doctrinal Buddhism’. That is, it includes all the officially sanctioned practices and beliefs of modern Theravada Buddhism.

Although both the position of the elbow in the scree plot and the contribution to fit indices of the third factor suggest it might be left out for the sake of parsimony, item loadings on this factor do lend themselves to a clear interpretation. Specifically, this factor loads positively on all cognitive (belief and interest) items, with the notable exception of the belief that nats have an influence in one’s life. It also loads negatively on the frequency of having the feeling that nats, weikza or the Buddha want to communicate something to one, which may suggest a trade-off between direct experience of supernatural agents on the one hand and an intellectual engagement with them on the other. Alternatively, because the scales and labels for belief and interest items were identical, but different from frequency items, this could be taken as a method factor; however, this interpretation fails to account for the fact that the belief in the intervention of nats in one’s life does not load on this factor. If it represents a cognitive dimension, and if there is a trade-off between intellectualising and interacting with supernatural agents, then this may better account for the failure of that item to load.

In attempting to interpret the two main dimensions, it would appear that instead of the distinctions implied by the hypotheses we set out to test, something more like the older distinction between ‘great’ and ‘little’ traditions may in fact be more apt. In Redfield’s [3] original conception, great and little traditions constituted two coexisting strata of village religion. In place of this definition, a variety of alternatives have been offered. Obeyesekere [26] claims that village religion is one composite whole, and thus we should think of the entirety of village religion as the ‘little tradition’, and the national or transnational religion of the literati as the ‘great tradition’. For Ames [29], Buddhism subdivides into a great tradition concerned with meditation and transcendence, and a little tradition concerned with merit-making for a better rebirth, while for animism, no distinction between great and little can be made.

Our data suggest that elements of each of these views are correct. The great tradition does, indeed, appear to correspond to the transnational religion promoted by the literati; but as Redfield argues, both great and little traditions coexist at the individual level, as two dimensions of religiosity. Meanwhile, perhaps more consistently with Obeyesekere’s argument, the little tradition is not animism, but appears to consist of all those elements of Buddhist religiosity which are not promoted by the official system. Great and little are simply two forms of Buddhist religiosity in which individuals can simultaneously partake.

### 3.3 Thanissaro’s scale of attitudes to Buddhism

The TSAB showed good internal consistency (Cronbach’s α = .875), albeit with a low mean inter-item correlation (r = .260). As a one-factor CFA model, it also showed good fit (CFI = .963; RMSEA = .038; χ^2^ (252, *N* = 2239) = 1048.009, p < 0.001; TLI = .959) with all loadings as expected.

A structural model (CFI = .933; RMSEA = .029; χ^2^ (1375, *N* = 2239) = 4002.150, p < 0.001; TLI = .930) was fitted combining the two-factor BBRS with the TSAB, and allowing the two religiosity trait factors to covary with the latter. The TSAB was strongly correlated with the second religiosity factor (r = .648, p < .001), but showed no significant correlation with the first factor. Given the doctrinally Buddhist content of the TSAB, this gives further support to the interpretation of the second factor as a form of ‘high’ or doctrinal religiosity, and the first factor as something independent from that.

A second structural model (CFI = .948; RMSEA = .026; χ^2^ (1368, *N* = 2239) = 3398.943, p < 0.001; TLI = .946) was fitted with the three-factor BBRS and the TSAB. Again, the TSAB was strongly correlated with the second BBRS factor (r = .609, p < .001), and showed no significant correlation with the first factor. However, TSAB was correlated with the third (cognitive) factor (r = .225, p < .001). This is perhaps unsurprising, considering the TSAB’s emphasis on doctrinal content.

In both models, the strong relationship between the TSAB and the second BBRS factor, as well as the lack of any relationship with the first BBRS factor, provide evidence for the concurrent validity of the second factor as a measure of ‘great tradition’ Buddhism, and for the suggestion that the first factor represents a distinct construct.

## 4. Study 2

This study aimed to test the great tradition/little tradition model in a confirmatory analysis, performed with a new sample. Demographic data were also collected to facilitate an exploratory analysis of correlates of the two traditions.

### 4.1 Methods

Participants were 3377 adult Facebook users in Myanmar, again excluding those from the original sample (n = 5853) who did not identify as Buddhists, did not identify Burmese as their mother tongue, or did not complete the religiosity scale. Targeted advertising was used to attract a larger proportion of respondents without a university education; however, the sample was still skewed toward those with at least some secondary education.

### 4.2 Results

CFA was carried out as in study 1, with sample weights calculated in the same way. Factor scores were estimated for further analysis using the maximum a posteriori method in Mplus [71].

The orthogonal, two-factor model above was employed in a CFA, resulting again in fit parameters just short of conventional thresholds (CFI = .946; RMSEA = .042; χ^2^ (405, *N* = 3377), p < 0.001; TLI = .942). Valences of all loadings were as predicted. Again, the three-factor model was a better fit (CFI = .970; RMSEA = .032; χ^2^ (395, *N* = 3377) = 1782.342, p < 0.001; TLI = .966).

Thus study 2 confirmed the great tradition/little tradition model of Burmese Buddhist religiosity in its three-factor form.

#### 4.2.1 Demographic correlates

Having established the adequacy of the three-factor model, and the near-adequacy of the two-factor model, we carried out an exploratory analysis of relationships between BBRS factor scores and various demographic variables. While these relationships did not form part of our original hypotheses, they are worth exploring, particularly given common suggestions that great tradition religiosity pertains to a more educated, urban elite, while little tradition religiosity is the purview of the rural, uneducated poor [3].

Table 4 shows Pearson correlations of the three BBRS factor scores with interval-level demographic variables. Great tradition adherence is positively related to age, number of offspring, and number of siblings, while the cognitive factor is negatively related to all three. The little tradition shows no relationship to any of these variables.

**Table 4:** Pearson correlations of BBRS factors with interval-level demographic variables (** = p < .01, * = p < .05).

	Age	Income (log)	Number of children	Number of siblings
Great tradition	.178**	-.017	.144**	.049*
Little tradition	-.004	-.005	-.002	.026
Cognitive	-.172**	-.047*	-.130**	-.060*

One-way ANOVAs were carried out in SPSS 25 [72] to compare the effects of nominal level demographic variables on the latent factors. Women scored significantly higher than men on the little tradition (F (1,3375) = 58.519, p < .001), and lower on cognitive religiosity (F (1,3375) = 17.776, p < .001), showing no difference on the great tradition. Those who reported having spent most of their lives in rural areas scored significantly higher on the great tradition (F (1,2494) = 10.683, p = .001) and on cognitive religiosity (F (1,2494) = 5.594, p = .018) than urbanites, but showed no difference on the little tradition.

For marital status, one-way ANOVA showed significant effects on the great tradition (F (4,2491) = 9.966, p < .001) and cognitive religiosity (F (4,2491) = 4.673, p = .001). Post-hoc comparisons using the Tukey HSD test indicated that those ‘in a relationship’ scored lower on the great tradition than all other categories (p < .005), while singletons scored lower than the widowed (p = .025). Cognitive religiosity was higher amongst singletons (p = .045) and those ‘in a relationship’ (p = .003) than for the married.

Finally, only the great tradition showed a significant effect of education (F (3,3373) = 6.521, p < .001). Post-hoc comparisons using the Tukey HSD test indicated this was entirely exhausted by a higher score amongst those with only a primary education than for those with a secondary (p = .002) or university education (p < .001).

## 5. Discussion

Our results reveal several surprising findings. First, contrary to claims for universal dimensions of religiosity, we do not find anything like such dimensions in the Burmese Buddhist case. While a weak third factor which may correspond to a cognitive dimension was present, it is debatable whether the additional variance explained by this factor is sufficient to justify the added complexity of the model. Thus while it may be that in other religious groups, dimensions such as intrinsic versus extrinsic religiosity are present, the Burmese Buddhist case appears to serve as a counterexample; and while a cognitive dimension may be present here, it is perhaps less prominent than has been suggested in other populations.

Second, and more importantly, having conducted the first psychometric test of the structure of Theravada religiosity, we find that none of the existing theoretical models adequately fit the data. For Ames [29], while little and great tradition Buddhism are concerned with outcomes in the afterlife and transcendence, respectively, ‘magical-animism’ exists to serve this-worldly purposes, improving the lot of practitioners in the present lifetime. Similarly, Spiro [30] classifies astrology as part of ‘apotropaic’ Buddhism (concerned with this-worldly aims), distinguishing this from, inter alia, the soteriological aims of the ‘esoteric Buddhism’ exemplified by the weikza. But in our analysis, the little tradition includes elements of all of these—apotropaic and soteriological, this-worldly and transcendent. Nor does the great tradition show any signs of subdividing into distinct domains such as ‘apotropaic’, ‘kammatic’ and ‘nibbanic’, contra Spiro.

However, while we do not dispute arguments for the conceptual and affective interdependency of elements of the religious system, our results suggest that Burmese Buddhist religiosity is not a one-dimensional, unipolar construct, but instead consists of more than one component. Moreover, contrary to arguments for a bipolar conception, these components are not in conflict with one another; instead, they constitute two orthogonal dimensions, with no apparent trade-off, and indeed no apparent mutual reinforcement either. However, our findings are not consistent with existing two-dimensional models, either; while these models have suggested either a Buddhism and an animism dimension, or a this-worldly and an other-worldly dimension, our data show that the dimensions in question do not correspond neatly to either one. These results call for a rethink of our understanding of the structure Theravada Buddhism.

The orthogonal relationship between the two dimensions is perhaps still more revealing. This structure clearly vindicates Ames’s [29] claim that there are two independent spectra in question. Moreover, it would suggest that the two forms of religiosity are not in conflict; adherence to the great tradition does not appear to demand any renunciation of the little tradition. Instead, the two appear to be independent. However, while this may indeed suggest they play complementary roles, the presence of the weikza in the first dimension again undermines Ames’s claim that this complementarity derives from a distinction between worldly and otherworldly concerns. Instead, if the elements of the little tradition have anything in common, it is that they are ‘esoteric’, which is just to say they appear to augment the austerity of high Buddhism with a worldview rich in supernatural agents and magic. Moreover, just as there appears to be no trade-off between doctrinal Buddhism and agentic views of nats and weikza, the agentic view of the Buddha, surprisingly enough, shows the same pattern.

### 5.1 The Buddha as a supernatural agent

The presence of the frequency of being communicated with by the Buddha in the little tradition dimension is intriguing. According to Theravada doctrine, while Gautama Buddha serves as an inspiration for contemporary Buddhists, he is no longer present as a conscious agent, having passed into a state of nirvana long ago. Although prayers may be directed at the Buddha, even beseeching him for help, this is taken to be nothing more than a way of focusing the practitioner’s mind, as the Buddha is not present to receive, let alone respond to, the prayers of his followers [73]. Nevertheless, practitioners worldwide frequently behave and speak as if the Buddha were a present, conscious agent [74]. Thus while the notion of the Buddha wanting to communicate something with one may be doctrinally incorrect, it is not an uncommon one.

Slone [74] argues that this contradictory conception of the Buddha, between the ‘ought’ and the ‘is’ of Buddhism, is entirely typical of religious believers across traditions, capable as they are of expressing correct doctrine when called upon to do so, but reverting to a more cognitively optimal conception of supernatural agents in other circumstances. Why should a living Buddha be more cognitively optimal than a non-available one? One potential explanation is that this is a more strongly anthropomorphic conception of the Buddha, in that it allows for the possibility of social interaction with him [75,76], a property which has been claimed to motivate anthropomorphism more generally [77]. This being the case, in order to enforce the counterintuitive notion of a non-agentive Buddha, doctrinal Theravada would have to rely on the strength of religious authorities and institutions dedicated to countering a takeover by more minimally counterintuitive ideas [78,79].

However, in that case, it would be reasonable to expect a conflict between affiliation with doctrine and a tendency to anthropomorphise the Buddha; that is, this item should be negatively related to the second dimension of the BBRS, which consists of items endorsed by Theravada doctrine (see below). As we found no such negative relationship, our findings may cast doubt on the notion that agentive conceptions of the Buddha represent a minimally counterintuitive ‘leakage’ in opposition to doctrinal enforcement.

Why, then, should this item load not negatively on the second dimension, but positively on the first? An alternative explanation might be found in contextual factors affecting the tendency to ascribe agency to supernatural beings. Van Leeuwen and van Elk’s [80] Interactive Religious Experience Model (IREM) hypothesises that personal beliefs about interactions with supernatural agents arise from an interaction between culturally transmitted concepts and agency intuitions which may themselves be facilitated by culturally constructed environments, some of which are more prone than others to produce experiences of supernatural agency. IREM predicts that religious traditions which provide more opportunities for individual experience of supernatural agency tend to produce greater personal belief in such interactions. The first BBRS dimension is, indeed, agentically rich; and it may be that individuals who regularly experience interactions with supernatural agents, such as nats and weikza, are similarly more strongly primed to experience the Buddha in an analogous way, as a social interactant.

### 5.2 Demographic considerations

A further point to consider is the relationship between the two dimensions and demographic variables. Contrary to expectations, we found no significant relationship with education, income, or rural versus urban life. Thus against official great tradition pronouncements, adherence to the little tradition does not appear to be the preserve of the undereducated or the rural; and nor does adherence to the great tradition appear to correspond to urban life or level of education. Instead, the only significant predictors found were age, in the case of the great tradition, and gender, in the case of the little tradition.

In the case of age, a number of plausible explanations are available. It is often claimed that older individuals become more involved in doctrinal Buddhism, because they have more time available to participate in calendrical rituals and related activities [30]. It may also be that with age, concerns with one’s next life become more salient, thus creating more incentives for merit-making. Alternatively, processes of secularisation and globalisation, which have accelerated markedly in Burma in recent years, may be more quick to take hold in the younger generation. Further investigation is necessary to adjudicate between these explanations.

As for gender, greater female affiliation with the little tradition may also be explicable in a number of ways. Our ethnographic observations, carried out in 2017 and 2018, would suggest that many nat festivals in particular are overwhelmingly attended by women; and spirit mediums we interviewed confirmed that nats are often now seen as the domain largely of women and gay men. Indeed, spirit mediums themselves tend either to be women or transgender men. Ethnographic evidence suggests that weikza followers are similarly constituted by a female majority [32]. It may be that while the great tradition is passed down through institutions, little tradition transmission relies more heavily on informal transmission, and that the primacy of mothers in the Burmese family puts women in a key node in the transmission chain [81]. Alternatively, there may be some functional distinction between great and little traditions such that the role played by the little tradition more closely aligns with the concerns of women in Burmese society.

It must be emphasised that both studies were limited not only to Facebook users with access to smartphones, but by their nature, to individuals willing and able to complete an online questionnaire. Given the unavailability of Internet access and computing resources in Myanmar until very recent years, it is perhaps unsurprising that both of our samples skewed heavily toward more educated people. Thus it cannot be assumed that these findings hold in groups with little or no education. Nevertheless, contrary to the claim that the little tradition is primarily associated with the uneducated, we found both great and little to be alive and well in our samples.

### 5.3 Conclusions

While this study has examined Burmese Buddhist religiosity, most debates in the literature have centred not around religiosity, but around religion. We are not of the view that the concept of religion is easily susceptible to clear definition, or indeed that it is necessarily analytically useful. It cannot be taken for granted that the relationship between individual religiosity and the social and cultural systems which might be labelled ‘religion’ is clear or obvious. Nevertheless, it is arguable that there must be some relationship; if, for example, Buddhism and animism constituted two distinct religious systems, we might expect individual affiliations to these two systems to vary along to distinct dimensions. Thus while our findings do not ultimately adjudicate between theoretical perspectives on Burmese religion itself, they do suggest that a new conceptual framework may be in order.

More broadly, our findings suggest that it may be fruitful for religiosity measures generally to test for the presence of both great tradition and little tradition forms of religiosity. Not only in Theravada Buddhist societies, but in other religious traditions, religiosity measures which concentrate solely on the great tradition run the risk of ignoring a crucial element of the religious system. While the locally specific nature of little traditions implies that such measures must of necessity be carefully adjusted to each context, the development of such measures would allow for the assessment of whether the surprising orthogonality found in the Burmese context applies more generally, and indeed, for an assessment of religiosity which does not exclude what is perhaps its most central component: orientation toward religion as a living, locally realised body of practice.
